# Giant Ovarian Cysts Treated by Single-Port Laparoscopic Surgery: A Case Series

**DOI:** 10.3389/fonc.2021.796330

**Published:** 2021-12-09

**Authors:** Lili Jiang, Xinyu Zhao, Yue Han, Kuiran Liu, Xinyue Meng

**Affiliations:** ^1^ Department of Obstetrics and Gynecology, Shengjing Hospital of China Medical University, Shenyang, China; ^2^ Department of Ultrasound, Shengjing Hospital of China Medical University, Shenyang, China

**Keywords:** giant, ovarian cyst, single-port laparoscopic surgery, case series, gynecologic oncology

## Abstract

**Background:**

Ovarian cysts are very common diseases of the female reproductive system. Giant ovarian cysts refer to the tumors with diameters greater than 10 cm. In recent years, due to the development of clinical diagnosis, imaging modalities, and the improvement of patients’ cognition of the diseases, the occurrence of giant ovarian cysts has become rare. The purpose of this study was to show a new operation method of single-port laparoscopy to treat giant ovarian cysts.

**Methods:**

We report a case series of five patients with giant ovarian cysts who underwent single-port laparoscopic surgery in the gynecology department of the Shengjing Hospital of China Medical University between June 2020 and March 2021. The inclusion criteria were ovarian cysts at least 20 cm in diameter, and cases when the tumor might be malignant were excluded.

**Results:**

The patients’ mean age was 26.2years. The most common clinical presentation was progressive abdominal distension. Median size of the cysts at imaging was 39.2 cm (range 21–63 cm). All patients underwent single-port laparoscopic surgery, and none of them converted to laparotomy. On final pathological reports, two cysts were serous cystadenomas, and three were mucinous cystadenomas. All patients recovered well and were discharged on time.

**Conclusion:**

Giant ovarian cysts can be treated by single-port laparoscopic surgery. In addition to the well-known advantages of laparoscopic surgery (e.g., small pelvic interference, fast postoperative recovery), it can also play the role of perfect cosmetic results, which has more advantages for young women.

## Introduction

Female pelvic cysts mostly come from the ovary and are asymptomatic when they are small. The symptoms appear when they reach enormous dimensions. Giant ovarian cysts (GOCs) are tumors larger than 10 cm in diameter or those cysts reaching above the umbilicus (1). Progressive abdominal distension, nonspecific diffuse abdominal pain, and organ compression (constipation, vomiting, and frequent urination) are the main clinical symptoms of ovarian cysts (2–4). Most giant ovarian cysts are treated by surgery. Surgical indications include a rapidly growing or symptomatic cyst, and when its malignant potential cannot be excluded (5). In the past, exploratory laparotomy was the most common surgical method, which had the advantage of minimizing the risk of an intraperitoneal implantation caused by cell overflow in case of an unexpected malignant transformation of the tumor. However, some giant ovarian cysts filled the abdominal cavity and superior reaching the xiphoid process. The abdominal incision reaching tens of centimeters long caused great trouble to patients, especially young women. In recent years, minimally invasive surgery has been widely used in the field of gynecology. Laparoscopy is the choice for most benign ovarian cysts, but the size of the cysts may be a limiting factor. Giant ovarian cysts increase the complexity and difficulty of laparoscopic surgery. Avoiding the leakage of cyst fluid has become a challenge (6). We report five cases of giant ovarian cysts treated by single-port laparoscopy. This method tries to ensure the oncologic safety while treating the disease. The aim of this study is to introduce a new, minimally invasive and effective surgical approach for the treatment of giant ovarian cysts.

## Materials and Methods

**Figure 1:**
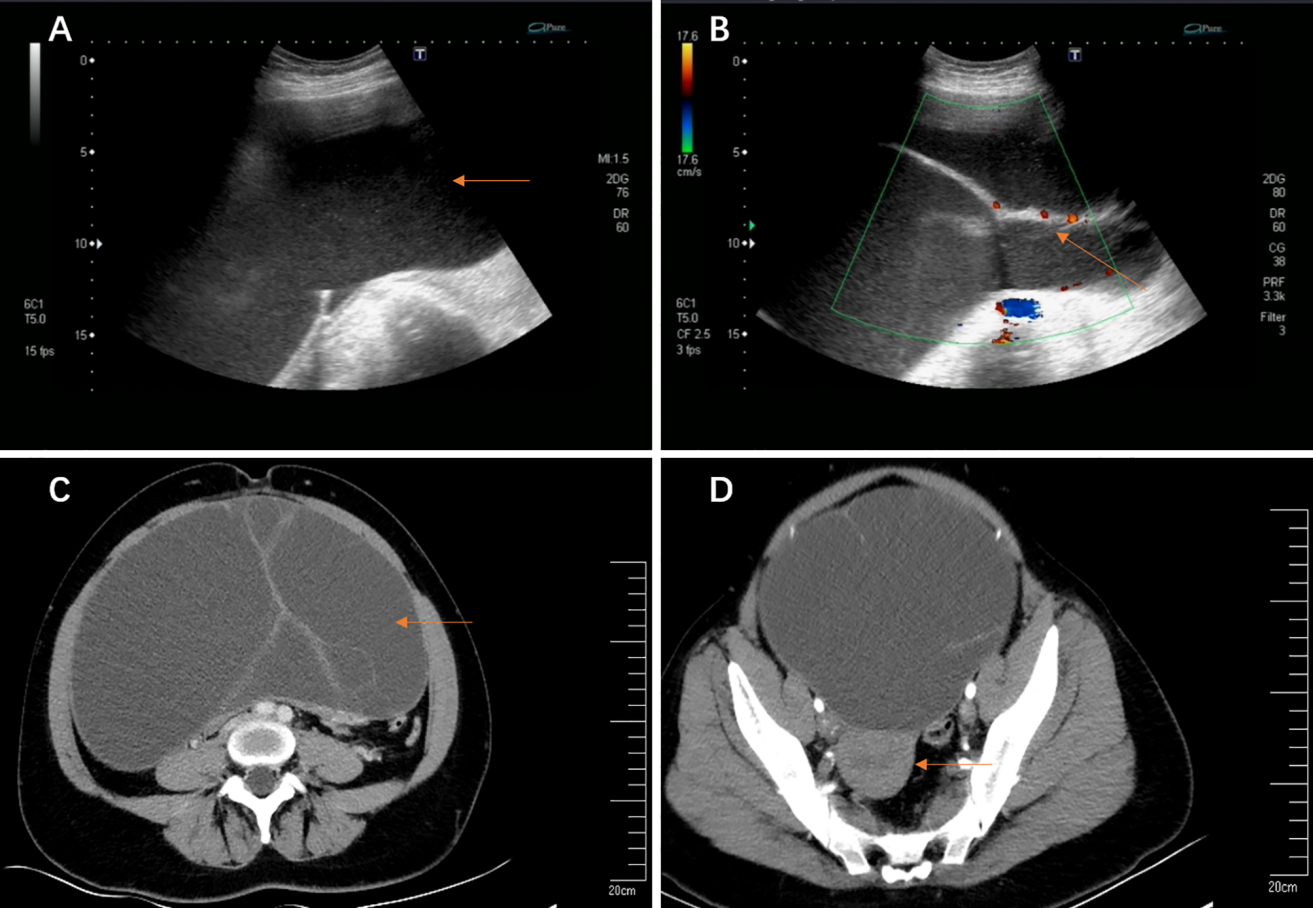
**(A, B)** Transvaginal ultrasound imaging. **(A)** A giant cyst in the abdominal and pelvic cavity (63.0 cm × 44.0 cm × 13.4 cm); **(B)** The blood flow signal detected at the separation; **(C, D)** Abdomen enhanced CT imaging. **(C)** A giant cyst with septums; **(D)** The uterus was pushed to the back of the pelvis by a giant cyst.

Five female patients with giant ovarian cysts who underwent single-port laparoscopic surgery between June 2020 and March 2021 were included from the gynecology department of the Shengjing Hospital of China Medical University. The study was approved by the China Medical University Research Ethics Committee. The inclusion criteria: ① All patients were diagnosed as giant abdominal cysts larger than 20 cm in diameter that tend to be benign by pelvic ultrasound, MRI or CT-scan before operation (**Figures 1A–D**). ② The patients had signed the informed consent. ③ The umbilicus was normal. Exclusion criteria: ① Conversion to open surgery or other surgical methods. ② Malignant transformation of cysts. ③ Severe medical system diseases which could not endure laparoscopic surgery. Five patients were confirmed by preoperative imaging (ultrasound, MRI or CT-scan) with giant abdominal masses at least 20 cm, mainly cystic, without obvious solid components, no abnormal increase in tumor markers obviously, showing that the ovarian cysts tend to be benign rather than malignant. Blood tumor markers (CA125, HE4, CA199, CA724, and CEA) were detected for each patient. The patients with complications were consulted in relevant departments to exclude surgical contraindications. In order to eliminate the influence of different surgeons’ experience on the surgical results, all patients were completed by a gynecologist who has experience in single-port laparoscopic surgery (author KL). Data were collected with operative time, intra- and post-operative complications, intracystic liquid volume, conversion to laparotomy, and the length of postoperative stay. Approximately 30 days after operation, the satisfaction of patients with abdominal scar was recorded. The score was 1–5 according to the wound recovery based on the subjective evaluation of the patients after the operation, which was the higher the score, the higher the satisfaction.

### Surgical Procedure

**Figure 2:**
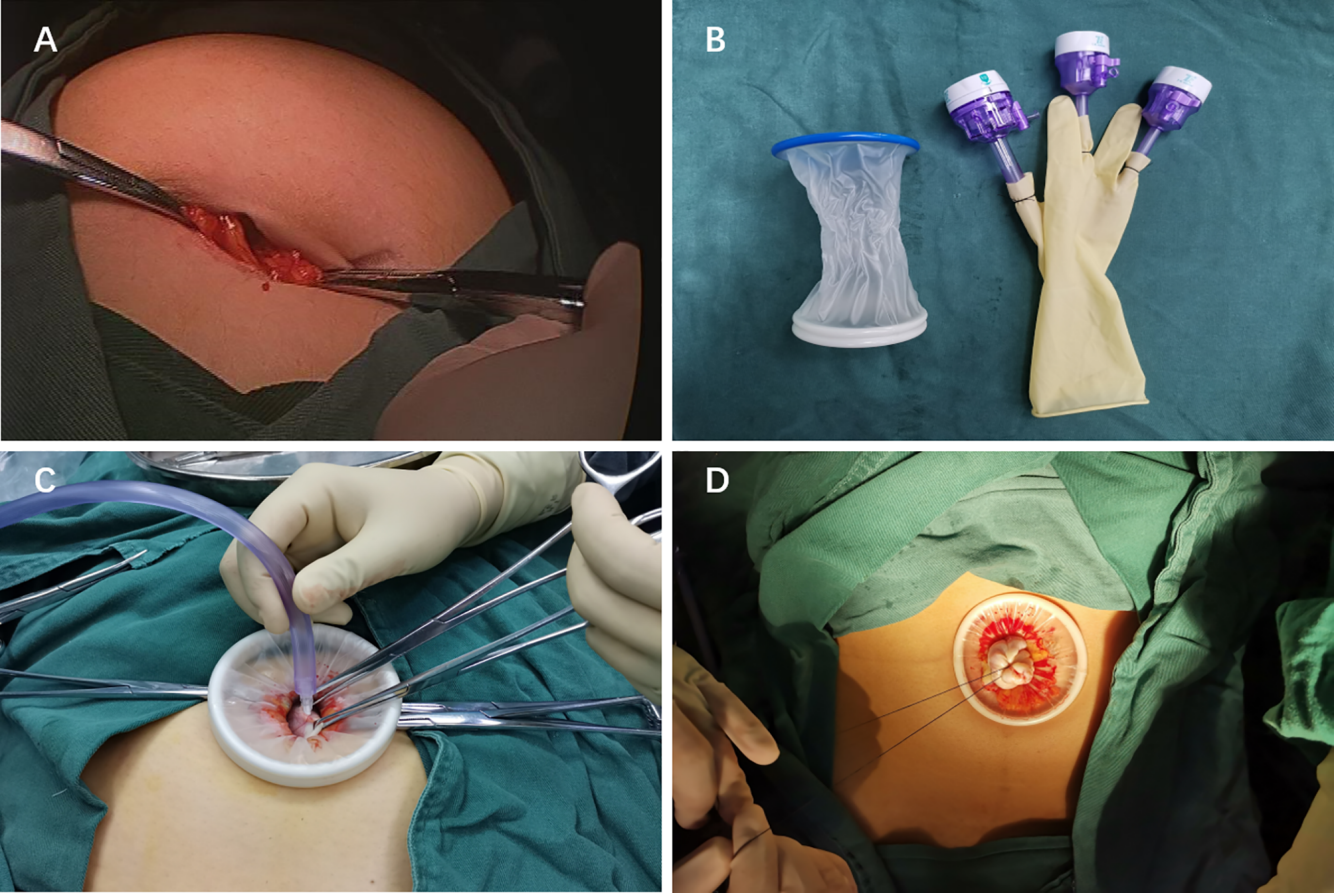
**(A)** The 2–3 cm longitudinal incision was made at the umbilicus. **(B)** Single-port laparoscopic access. **(C)** A syringe needle connected with a suction device to suck out the liquid in the cyst. **(D)** Ligate the incision in order to avoid the leakage of cyst fluid.

The patients received standardized preoperative nursing preparation and general anesthesia. Single-port laparoscopic surgery was performed using the following techniques. After partial eversion of the umbilicus, a 2–3 cm longitudinal incision was made at the umbilicus (**Figure 2A**). The umbilical incision was lifted, the skin and subcutaneous tissue were incised layer by layer, and the peritoneum was incised after confirming that there was no intestinal adhesion under the incision. The disposable incision protection sleeve (Lookmed, Jiangsu, China) was placed in the incision, the inner ring was placed in the abdominal cavity, and the outer ring was left to the abdominal wall to form a single-port laparoscopic approach access (**Figure 2B**). A giant cyst appeared under the incision and the inside of the cyst was mainly liquid. In order to prevent the adverse effects of sudden drop of abdominal pressure on patients, we used a syringe needle connected with a suction device to slowly suck out the liquid in the cyst (**Figure 2C**). If the cyst divided into several septums, we suck out the liquid in one septum and then used the instruments to lift the wall of the cyst to prevent the leakage of the liquid in the cyst. We changed another septum and continued to suck out the liquid to reduce the pressure of the cyst. When the liquid was sucked out completely, we used silk thread to ligate the incision and returned the cyst to the abdominal cavity (**Figure 2D**).

**Figure 3:**
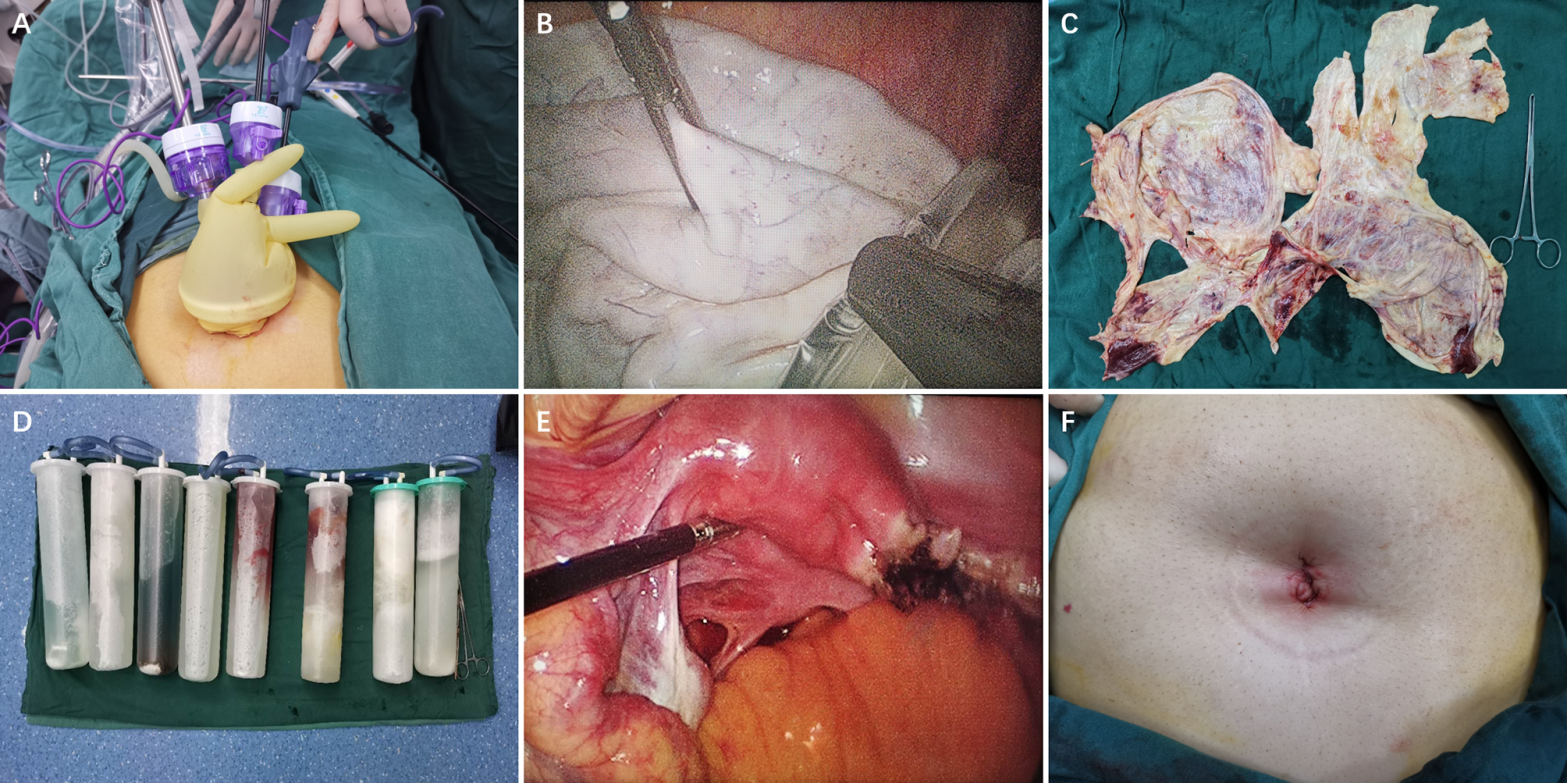
**(A)** The instruments enter the abdominal cavity through single-port laparoscopic access. **(B)**The excised tissue was put into an endopouch specimen retrieval bag under laparoscope. **(C)** The wall of a giant cyst removed through the navel. **(D)** Intraoperative suction of intracapsular fluid. **(E)** Unilateral salpingo-oophorectomy by laparoscope. **(F)** A cosmetic suture of the single-port laparoscopic incision in patients’ navel.

A sterile glove was connected with the outer ring. The thumb of the glove was cut, and 10 mm trocar (Dike, Guangzhou, China) was placed as the access of a scope and laparoscopic instruments. In order to prevent air leakage and loosening at the joint, a No. 7 silk thread was used to fix and was tied tightly, and the 5 mm (Dike, Guangzhou, China) trocars were inserted into the other two fingers as the instrument port. This is a self-made simple laparoscopic single-port (**Figure 3A**). This is a cost-saving advantage for the patients without affecting the operation.

Carbon dioxide was injected at a pressure of 13 mmHg and a rigid 30° 5-mm laparoscope was inserted (Karl Storz, Tutlingen, Germany). A 30° laparoscope is a better choice because it provides a wide field of vision. Then the standard laparoscopic surgery was performed. Giant ovarian cysts were removed from the umbilicus using an endopouch specimen retrieval bag (Wellead, Guangzhou, China) (**Figures 3B, C** **)**.

## Results

The study consisted of five female patients and data are shown in **Tables 1**, **2**. The mean age of the operated patients was 26.2 years (range, 19–34 years). The BMI of the five patients ranged from 16.8 to 31.2. According to the BMI calculation results, one patient was thin (patient 3), one normal (patient 4), two obese (patients 1 and 2), and one overweight (patient 5). Coincidentally, all of the patients had no history of gravidity, parity, and previous abdominal operations.

**Table 1:** Clinical characteristics of the five patients.

Characteristics	Age	BMI	Gravidity	Parity	No. of previous abdominal operations
**1**	23	26.3	0	0	0
**2**	34	25.6	0	0	0
**3**	19	16.8	0	0	0
**4**	25	18.8	0	0	0
**5**	30	31.2	0	0	0

**Table 2:** Surgical outcomes of the five patients.

Patient	Age	Cyst size(cm)	Operative time(min)	Fluid volume in cyst (ml)	Intra-op. blood loss (ml)	Post-op. stay (d)	Conversion to laparotomy	Histology	Post-op. complications	Satisfaction with abdominal scar
**1**	23	32	100	7,000	20	5	No	Serous cystadenoma	No	4
**2**	34	63	75	16,000	20	5	No	Mucinous cystadenoma	No	5
**3**	19	21	37	3,500	10	5	No	Mucinous cystadenoma	No	4
**4**	25	23	82	4,000	50	4	No	Serous cystadenoma	No	4
**5**	30	57	132	13,000	30	6	No	Mucinous cystadenoma	No	5
**Mean**	26.2	39.2	85.2	8,700	26	5	–	–	–	4.4

Surgical outcomes are shown in **Table 2**. The most common symptom was progressive abdominal distension (patients 1, 2, 4, and 5), several of which were accompanied by abdominal pain (patients 1, 2, and 5). No obvious abdominal distension occurred in patient 3, mainly due to palpation of abdominal mass. All patients were diagnosed by imaging, ultrasound, MRI or CT-scans. Median size of the cysts at imaging was 39.2 cm (range 21–63 cm), while the maximum was 63.0 cm with the superiors reaching the sword (patients 2). In particular, there were much comorbidities in patient 2. Hypertension occurred 17 years ago. Now oral antihypertensive drugs are used to control blood pressure, and the blood pressure is controlled at 130/80 mmHg. In 2014, she suffered from cerebral thrombosis. The specific location is unknown. She felt numb on the right side of the body at the time of onset, which was improved after a conservative treatment but was left hemiplegic of the right limb. We consulted the anesthesiology, cardiology, and neurology departments before operation to evaluate the safety of the operation and eliminate the operation contraindications. Based on the patient’s age and personal will, we decided to perform single-port laparoscopic exploration after discussion.

Four of the five patients presented with normal blood tumor markers. One patient presented with an elevated CA125 of 70.78 (normal range 0–35 mIU/ml) and CA-724 of 8.94 (normal range 0–6.9 mIU/ml) (patient 3). In the postoperative reexamination, the blood tumor markers gradually returned to normal. All patients underwent single-port laparoscopic surgery, and no one converted to laparotomy. Intraoperative suction of intracapsular fluid was in the range of 3,500–16,000 ml (**Figure 3D**).The average volume was 8,700 ml. Four patients underwent unilateral adnexectomy, and one patient an ovarian cystectomy (**Figure 3E**). We had a cosmetic suture of the single-port laparoscopic incision in the patients’ navels (**Figure 3F**). The average operative time was 85.2 min (range 37–132 min). Neither extravasation of cystic fluid and nor decompression syndrome happened due to the gradual reduction of cystic pressure. Mean blood loss was 26 ml (range 10–50 ml). The average time of hospitalization after operation was 5 days, and such operative method did not increase the post-operative stay. All patients recovered well, and no complications related to the operation occurred. On final pathological reports, two cysts were serous cystadenomas, and three were mucinous cystadenomas. There was no borderline tumor or epithelial ovarian cancer in any of the ovarian cysts operated, but one case reported an active cell proliferation, which should be reexamined. All the patients were satisfied with the abdominal scar 30 days after the operation.

## Discussion

Female pelvic cysts are a very common gynecological disease in women, most of which come from the ovary. During their lifetime, it is assumed that about 7% of women experience a symptomatic cyst worldwide (7). The clinical manifestations appear when the cysts reach enormous dimensions. Giant ovarian cysts (GOCs) are tumors larger than 10 cm in diameter (1). Due to improved imaging techniques, giant abdominal cysts have increasingly become rare. The patients can present with rare complications such as torsion, intestine obstruction, and hydronephrosis in addition to causing non-specific abdominal distension, pain, nausea, and vomiting and changes in defecation habits (8–11). As the nonspecific clinical manifestations of giant ovarian cysts, the differential diagnoses include the giant cysts arising from other intra-abdominal organs (e.g., gastrointestinal, urological, or lymphatic) (12).

The treatment of ovarian cysts depends on the patient’s age, the size of the cyst, and its histopathological feature. Excision of the intact cysts for histology is the gold standard (13). Most giant ovarian cysts are benign and are generally treated by surgical excision either by a cystectomy or a salpingo-oophorectomy (14). It is of utmost importance to exclude any possibility of malignant tumor before operation (15). The SRU guidelines propose that within an ovarian or adnexal cystic lesion, multiple thin septations (<3 mm) or an avascular, solid non-hyperechoic nodule are indeterminate characteristics, often found in benign neoplasms. If the cyst is more than 10 cm in size or continues to have indeterminate findings, surgical evaluation should be considered (16).

In the past, the resection of the cystic mass by an exploratory laparotomy was the preferred management strategy (9). But for laparotomy of benign giant cysts, the huge incision caused trouble to the patients (especially young patients). A study showed that with the development of advanced technology, it was feasible to use laparoscopic surgery to remove giant ovarian cysts on the basis of selecting suitable patients and laparoscopic experts (17). Recently, laparoscopic-assisted excision of these giant cysts had been reported in several literatures (6, 18, 19). Avoiding the leakage of cyst fluid has become a challenge in laparoscopic surgery for treating giant ovarian cysts.

In recent years, single-port laparoscopic surgery has become a hot spot as it uses the natural pores of the navel to hide the surgical incision and has the characteristics of perfect cosmetic results and fast postoperative recovery. In our study, we used a single-port laparoscope to perform surgery on a slightly larger incision at the umbilicus, which exposed the visual field better and avoided the exudation of liquid in the giant cysts. In order to avoid the impact of sudden drop of intraperitoneal pressure on the patients, we used the method previously described to slowly reduce the fluid in the giant cysts. Facts had proved that this method is effective, and these patients did not appear to have related uncomfortable symptoms. We use the wound protector–retractor to protect the incision and reduce the risk of cell spillage. The endopouch specimen retrieval bag was used to take out the specimen after the resection of the diseased tissue, which reduced the potential risks of the leakage of cells and residual cystic fluid. These measures ensured the safety of the operation. Although giant ovarian cysts are larger than 10 cm in diameter, we still selected cysts larger than 20 cm in diameter for study, which are rarer in clinical practice. We analyzed the general information and surgical outcomes of these patients and found that a single-port laparoscopic surgery did not increase the adverse prognosis of patients. On the contrary, a minimally invasive surgery and perfect cosmetic results accelerated the recovery and satisfaction of patients.

Despite the advantages of single-port laparoscopic surgery, not all giant ovarian cysts are suitable for this type of surgery. We need to evaluate the patient’s condition before undergoing an operation rigorously, and it is very important to exclude any possible malignant tumors before operation. Forming an operation triangle in a single-port laparoscopic surgery is difficult due to its limited operation space, relatively concentrated instruments, and mutual interference which propose higher demands to the surgeon. It is necessary for us to improve the safety of surgery through more research.

## Conclusion

In the treatment of giant ovarian cysts, it is safe and feasible to perform single-port laparoscopic surgery through the strict screening of suitable patients. This operation method has the same advantages of traditional laparoscopy, and it ensures the safety of operation as far as possible and perfectly improves the cosmetic results, which are particularly important for young women.

## Data Availability Statement

The original contributions presented in the study are included in the article/supplementary material. Further inquiries can be directed to the corresponding authors.

## Ethics Statement

The studies involving human participants were reviewed and approved by China Medical University Research Ethics Committee. The patients/participants provided their written informed consent to participate in this study. Written informed consent was obtained from the individual(s) for the publication of any potentially identifiable images or data included in this article.

## Author Contributions

LJ conducted a thorough literature review and was the major contributor in writing the manuscript. YH and XZ were responsible for reviewing the literature and collecting the patients’ information. KL was responsible for performing the surgery. XM was responsible for providing the figures, screening patients and for performing ultrasound examinations. All authors contributed to the article and approved the submitted version.

## Funding

This research received the support from a “Scientific research funding project of the Liaoning Provincial Department of Science and Technology (No.2020JH2/10300050)”.

## Conflict of Interest

The authors declare that the research was conducted in the absence of any commercial or financial relationships that could be construed as a potential conflict of interest.

## Publisher’s Note

All claims expressed in this article are solely those of the authors and do not necessarily represent those of their affiliated organizations, or those of the publisher, the editors and the reviewers. Any product that may be evaluated in this article, or claim that may be made by its manufacturer, is not guaranteed or endorsed by the publisher.

## Abbreviations

GOCs, Giant ovarian cysts; MRI, Magnetic Resonance Imaging; CT, Computerized Tomography; CA125, Carbohydrate antigen-125; HE4, Human Epididymis Protein 4; CA199, Carbohydrate antigen-199; CEA, Carcinoembryonic antigen.
